# Fingolimod additionally acts as immunomodulator focused on the innate immune system beyond its prominent effects on lymphocyte recirculation

**DOI:** 10.1186/s12974-017-0817-6

**Published:** 2017-02-23

**Authors:** Katja Thomas, Tony Sehr, Undine Proschmann, Francisco Alejandro Rodriguez-Leal, Rocco Haase, Tjalf Ziemssen

**Affiliations:** Center of Clinical Neuroscience, Department of Neurology, Carl Gustav Carus University Hospital, University of Technology Dresden, Fetscherstr. 74, 01307 Dresden, Germany

**Keywords:** Innate immunity, Dendritic cells, Antigen-presenting cells, Sphingosine-1-phosphate-directed therapies, Multiple sclerosis

## Abstract

**Background:**

Growing evidence emphasizes the relevance of sphingolipids for metabolism and immunity of antigen-presenting cells (APC). APCs are key players in balancing tolerogenic and encephalitogenic responses in immunology. In contrast to the well-known prominent effects of sphingosine-1-phosphate (S1P) on lymphocyte trafficking, modulatory effects on APCs have not been fully characterized.

**Methods:**

Frequencies and activation profiles of dendritic cell (DC) subtypes, monocytes, and T cell subsets in 35 multiple sclerosis (MS) patients were evaluated prior and after undergoing fingolimod treatment for up to 24 months. Impact of fingolimod and S1P on maturation and activation profile, pro-inflammatory cytokine release, and phagocytotic capacity was assessed in vitro and ex vivo. Modulation of DC-dependent programming of naïve CD4+ T cells, as well as CD4+ and CD8+ T cell proliferation, was also investigated in vitro and ex vivo.

**Results:**

Fingolimod increased peripheral slanDC count—CD1+ DC, and monocyte frequencies remained stable. While CD4+ T cell count decreased, ratio of Treg/Th17 significantly increased in fingolimod-treated patients over time. CD83, CD150, and HLADR were all inhibited, but CD86 was upregulated in DCs after incubation in the presence of fingolimod. Fingolimod but not S1P was associated with reduced release of pro-inflammatory cytokines from DCs and monocytes in vitro and ex vivo. Fingolimod also inhibited phagocytic capacity of slanDCs and monocytes. After fingolimod, slanDCs demonstrated reduced potential to induce interferon–gamma-expressing Th1 or IL-17-expressing Th17 cells and DC-dependent T cell proliferation in vitro and in fingolimod-treated patients.

**Conclusions:**

We present the first evidence that S1P-directed therapies can act additionally as immunomodulators that decrease the pro-inflammatory capabilities of APCs, which is a crucial element in DC-dependent T cell activation and programming.

## Background

Multiple sclerosis (MS) is a chronic inflammatory disease of the central nervous system (CNS) that is mediated mainly by activated pro-inflammatory CD4+ T helper (Th) cells and cytotoxic CD8+ T cells [1, 2]. Growing evidence is available that suggest a role for antigen-presenting cells (APC) in the pathogenesis of MS via their extraordinary capacity for inducing and expanding pro-inflammatory T cell populations [3, 4]. In particular, dendritic cells (DC) play a crucial role in regulating the balance between encephalitogenic and tolerogenic immunity in MS [5]. We recently demonstrated the presence of 6-sulfo LacNAc+ (slan) DCs, which are the major pro-inflammatory and most potent T cell-activating DC populations, in active inflammatory MS lesions. SlanDCs represent a new potential link between innate and adaptive immunity in MS and are specifically modulated by different MS therapies [6, 7]. As such, future treatments should include targeted modulation of selective DC and APC functions [8, 9].

Fingolimod (FTY) is the first approved oral therapy for highly active relapsing remitting (RR) MS. Fingolimod exerts its effect via modulation of the sphingosine-1-phosphate (S1P)-receptor (S1PR) [10, 11]. Extensive data on the mechanism of action of fingolimod demonstrate its principal effects on T and B cell trafficking via impairment of S1PR1-mediated recirculation, which results in significantly reduced lymphocyte egress from lymphoid tissues into the general circulation [12]. In addition to the effects on T and B cells, modulation of the innate immune system, including actions on DCs, have been proposed [13–17]. Sphingolipids and their G-protein-coupled receptors appear to play an important role in the modulation of the innate immune system. Additionally, all of the known sphingolipid receptor-subtypes (S1PR1-S1PR5) are apparently involved in the modulation of function and metabolism of APCs [13, 18, 19]. Although the circulation of APCs is not primarily regulated by the S1P-system, FTY and its active metabolite FTY-phosphate (FTYP) appear to affect APC migration into lymph nodes and tissues possibly via modulation of inflammatory chemokines [18, 20–22]. However, human data on effects of FTY on APC subsets in MS patients are rare, and the detailed impact on pro-inflammatory potential and DC-dependent T cell regulation lack detailed understanding.

To gain novel insights into immunomodulatory effects of FTY on innate immunity beyond the established effects on lymphocyte recirculation, we investigated the FTY-stimulated ex vivo and in vitro modulation of frequency and function of slanDC (the most potent pro-inflammatory DC population) to evaluate the impact of FTY on inflammatory and T cell regulatory properties. Here, we present data on the impact of FTY on the inflammatory properties of slanDCs and classical APCs via in vitro and ex vivo analyses of FTY-treated MS patients.

## Methods

### Patients and controls

Blood samples of 35 RRMS patients diagnosed according to the McDonald criteria were used to evaluate immunomodulatory effects on APC during FTY treatment (Table 1). Blood samples were drawn prior to and during FTY treatment up to 24 months. Further blood samples were collected of ten untreated RRMS patients with stable disease course compared to ten RRMS patients with stable disease after 12 months of FTY therapy to perform additional ex vivo analyses. Blood of healthy donors was collected for in vitro analyses.

**Table 1:** Patient characteristics

Patient Nr.	Sex	Age	Disease duration [years]	Pretreatment	EDSS	Stable disease course
1	F	23	3	Interferon-beta	1.5	Yes
2	F	54	9	None	2	Yes
3	M	28	4	Interferon-beta	1.5	Yes
4	M	33	14	Natalizumab	4	Yes
5	F	42	14	Glatiramer acetate	3	Yes
6	F	53	16	Interferon-beta	3.5	Yes
7	F	47	3	Interferon-beta	3	Yes
8	F	47	7	Interferon-beta	2	Yes
9	F	41	11	Interferon-beta	6	Yes
10	F	46	15	Natalizumab	4	Yes
11	F	42	2	None	1	Yes
12	F	19	2	None	1.5	Yes
13	F	30	11	Natalizumab	2	Yes
14	M	35	3	Glatiramer acetate	2	Yes
15	M	27	3	Glatiramer acetate	2	Yes
16	F	44	4	Interferon-beta	2.5	Yes
17	F	27	11	Natalizumab	2	No
18	F	29	15	Natalizumab	6	Yes
19	F	24	11	None	1	Yes
20	F	47	6	Glatiramer acetate	3	Yes
21	F	45	11	Glatiramer acetate	1.5	Yes
22	M	42	3	Glatiramer acetate	4	Yes
23	F	44	10	Interferon-beta	1.5	Yes
24	M	38	3	Glatiramer acetate	1.5	Yes
25	M	26	4	Interferon-beta	1.5	No
26	F	42	4	Glatiramer acetate	2	Yes
27	M	29	2	Glatiramer acetate	2	Yes
28	F	35	2	Glatiramer acetate	3	Yes
29	M	41	1	None	2	Yes
30	M	30	6	Interferon-beta	1.5	Yes
31	M	28	1	None	1.5	Yes
32	M	44	10	Interferon-beta	4	Yes
33	F	45	10	Interferon-beta	4	Yes
34	M	41	19	Natalizumab	3	Yes
35	F	33	8	Glatiramer acetate	2.5	Yes

Sex, age at FTY start, time from disease onset to FTY start, pretreatment, baseline EDSS, and disease course (stable versus not stable) are depicted

All experiments were approved by the institutional review board of the University Hospital of Dresden. All donors gave their written informed consent.

### Flow cytometric analysis

Preparation of blood cells and analysis by fluorescence-activated cell sorting (FACS) have been performed by a previously validated protocol defined by standard operating procedures (SOPs): Peripheral blood mononuclear cells (PBMCs) were prepared by Ficoll–Hypaque (Biochrom, Berlin, Germany) density centrifugation. Cell surface staining was performed by using fluorescence-labeled anti-CD3, anti-CD4, anti-CD8, anti-CD14, anti-CD19, anti-CD40, anti-CD80, anti-CD83, anti-CD86, anti-CD150, anti-HLADR (BD Biosciences, Heidelberg, Germany), anti-BDCA1, anti-slan, or anti-CD39 (Miltenyi Biotec, Bergisch Gladbach, Germany) according to the manufacturer’s instructions. Negative controls included directly labeled or unlabeled isotype-matched irrelevant antibodies (BD Biosciences). For further characterization of intracellular markers, PBMCs were suspended in culture medium consisting of RPMI 1640 (Biochrom), 5% human AB serum (CC pro, Neustadt, Germany), 2 mM l-glutamine, 100 U/ml penicillin, and 100 μg/ml streptomycin (Biochrom). Analysis of T regulatory cells (Treg cells) was performed directly, whereas Th17 cells were stimulated with 10 ng/ml phorbol myristate acetate (PMA, Sigma-Aldrich, Steinheim Germany) and 1 μg/ml ionomycin (Sigma-Aldrich) in the presence of 0.2 μM Monensin (Biomol, Hamburg, Germany) for 6 h prior to analysis. For intracellular characterization of IL-17, CD154, and FoxP3, cells were fixed with fresh prepared fixation concentrate and permeabilized with wash-permeabilization concentrate (Fixation/Permeabilization Buffer Set, eBioscience). Subsequently, cells were stained using fluorescence labeled anti-IL-17 (BioLegend, London, UK), anti-CD154 and anti-FoxP3 antibody (both Miltenyi Biotec), or isotype-matched irrelevant antibody (BD Biosciences). After the staining procedure, cells were evaluated on a FACScan Calibur (BD Bioscience). Exact preparation of the cells, staining protocol, and procedure as well as adjustment and compensation of the FACScan was established prior to first analysis of samples. Complete blood cell count was performed additionally to FACS analysis. No patients with lymphopenia <0.2 GPt/l or lower medical drug possession rate >95% during fingolimod treatment were included to guarantee reliable data.

### Immunomagnetic cell sorting

Isolation of slanDCs was performed as described previously [6]. PBMCs were incubated with M-DC8 hybridoma supernatant containing 10 μg/ml of antibody and additional rat anti-mouse IgM paramagnetic microbeads (Miltenyi Biotec). Cells were sorted on two columns via the autoMACS device (Miltenyi Biotec, Bergisch Gladbach, Germany). CD1 + DC were sorted by depletion of CD19+ cells first, followed by positive selection of BDCA1+ using immunomagnetic separation according to the manufacturer’s instructions (Miltenyi Biotec, Bergisch Gladbach, Germany). CD14+ monocytes were isolated by positive selection, and CD4+ T cells, CD8+ T cells, and naive CD45RA + CD4+ T cells were isolated by depletion using immunomagnetic separation (Miltenyi Biotec, Bergisch Gladbach, Germany). The purity of the isolated cell populations was >95% as always assessed by flow cytometry afterwards.

### Cytokine assay

Sorted slanDCs, CD1 + DCs, and monocytes of untreated or FTY-treated patients were cultured for 24 h. For the last 18 h, lipopolysaccharide (LPS, Sigma Aldrich) was added to stimulate cytokine release by TLR4 activation; unstimulated cells served as control. Additionally, cells of healthy controls and FTY-treated patients were maintained in the presence or absence of 30 ng/ml FTY, 30 ng/ml FTYP (Caltag, Buckingham, UK), or 20 or 200 nM S1P (Sigma Aldrich) in culture before LPS was added. Supernatants were collected, and the concentration of tumor necrosis factor alpha (TNF-alpha), IL-1beta, IL-6, IL-12, and IL-23 was determined using a commercial ELISA kit (BD Biosciences) according to the manufacturer’s instructions.

### Maturation and activation profile

Sorted slanDCs, CD1+ DCs, and monocytes of healthy controls or FTY-treated patients were cultured in the presence or absence of 30 ng/ml FTY or 30 ng/ml FTY-phosphate or 20 or 200 nM S1P in vitro. Cells were collected and characterized with regard to surface activation and maturation markers by staining with fluorescence labeled anti-CD40, anti-CD80, anti-CD83, anti-CD86, anti-CD150, and anti-HLA-DR (BD Biosciences). Cells were evaluated on a FACScan Calibur.

### DC-depending T cell proliferation and programming

SlanDCs or CD1 + DCs of healthy controls were cultured with or without 3 or 30 ng/ml FTY or FTYP for 6 h and washed with phosphate-buffered saline (PBS, Sigma Aldrich). To evaluate T cell proliferation, allogeneic CD4+ T cells or CD8+ T cells were labeled with carboxyfluorescein-di-acetate-*N*-succinimidylester (CFSE, Molecular Probes, Eugene, USA) at a final concentration of 0.3 μM. Treated and untreated DCs (1 × 10^4^ cells/well) were co-cultured with CFSE-labeled allogeneic CD4+ T cells or CD8+ T cells (1 × 10^5^ cells/well) for 4 days. Cells were harvested, and proliferation was calculated by CFSE-incorporation by flow cytometry and quantified by cell division index (CDI). For ex vivo analyses, slanDCs of FTY-treated patients compared to healthy controls were co-cultured with CFSE-labeled allogeneic CD4+ or CD8+ T cells of the same healthy donor to compare different potentials to induce T cell proliferation. To assess direct effects of FTY or FTYP on T cells, sorted CFSE-labeled CD4+ T cells or CD8+ T cells of healthy donors or FTY-treated patients were cultured in the presence of 5 μg/ml human anti-CD3 and 1 μg/ml human anti-CD28 (both BD Bioscience) without or with FTY or FTYP for 4 days. CFSE-incorporation was evaluated and counted as described above.

To evaluate DC-dependent T cell programming, FTY or FTYP pretreated and untreated slanDCs or CD1 + DC (1 × 10^4^ cells/well) of healthy controls were co-cultured with allogeneic naïve CD45RA + CD4+ T cells (1 × 10^5^ cells/well) in the presence of LPS for 8 days. Thereafter, T cells were stimulated with 10 ng/ml PMA and 1 μg/ml ionomycin in the presence of 0.2 μM monensin for 4 h. For intracellular characterization of IFN-gamma, IL-17 and IL-4 production, cells were fixed with freshly prepared ice-cold 4% paraformaldehyde (Merck) and permeabilized with 0.1% saponin (Merck) in PBS containing 1% fetal calf serum (FCS, Biochrom). Subsequently, cells were stained using fluorescence-labeled anti-IFN-gamma, anti-IL-17, and anti-IL-4 antibody or isotype-matched irrelevant antibody (BD Biosciences). After the staining procedure, cells were evaluated on a LSR Fortessa (BD Bioscience). For ex vivo analyses, slanDCs of FTY-treated patients compared to healthy controls were co-cultured with naïve CD45RA+ CD4+ T cells of the same healthy donor to compare different potential in T cell programming. To compare impact of FTY or FTYP on potential of polarization directly on T cells, naïve CD45RA+ CD4+ T cells stimulated with 5 μg/ml human anti-CD3 and 1 μg/ml human anti-CD28 treated without or with FTY or FTYP served as control. Differentiation into Th1 T cells was induced by adding 10 ng/ml human IL-12 and 10 μg/ml human anti-IL4, whereas Th2 differentiation was ensured by adding 10 ng/ml human IL-4 and 10 μg/ml human anti-IFN-gamma (all R&D Systems). After 8 days of cell culture, T cells were prepared and analyzed as described above.

### Phagocytosis assay

Sorted slanDCs, CD1+ DCs, and CD14+ monocytes of healthy donors were maintained for 12 h in the presence or absence of 3 ng/ml or 30 ng/ml of FTY or FTYP in culture. To analyze, phagocytotic ability cells were treated with 1 μm carboxylate-modified yellow–green fluorescent FluoSpheres beads (Thermo Fisher Scientific, MA, USA) for 60 min at 37 °C. After cells were washed with PBS, incorporation of beads was evaluated by FACScan Calibur.

### Apoptosis assay

Sorted slanDCs, CD1 + DCs, and CD14+ monocytes of healthy donors were cultured for 24 or 48 h in the presence or absence of different concentrations of FTY or FTYP (3 ng/ml; 30 ng/ml). Annexin was measured using a FITC-labeled antibody (BD Bioscience) to determine apoptosis at early stage, and APC-labeled fixable viability dye staining (BD Bioscience) was used to evaluate apoptosis at late stage characterized by DNA fragmentation. After, staining cells were analyzed by FACScan Calibur.

### Statistical analysis

For repeated measure testing, repeated measure analysis of variance (ANOVA) with Bonferroni’s correction for compared pairs was used. Analyses with multiple comparisons but not repeated testing were evaluated by ANOVA with Bonferroni’s correction. Analyses without multiple testing were assessed by Student’s *t* test. Values of *p < 0.05, **p < 0.01, and ***p < 0.001 were considered significant.

## Results

### Increase in slanDC frequency in comparison to T cell frequency changes in peripheral blood compartment during long-term FTY treatment

**Fig. 1:**
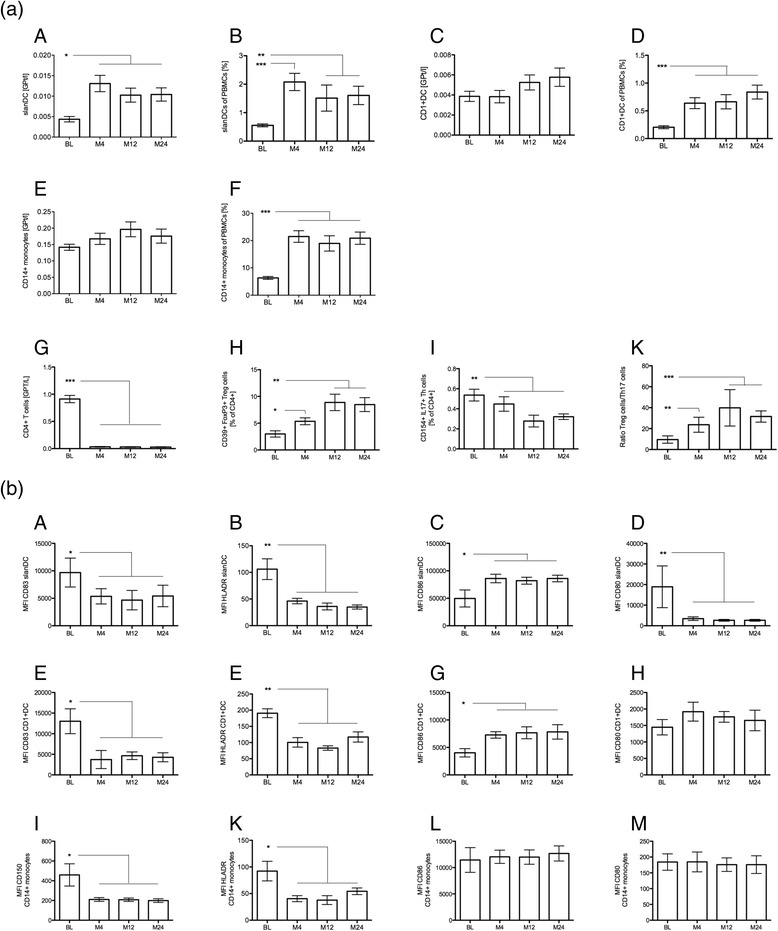
APC and T cell count in FTY-treated RRMS patients. **a** Relative and absolute cell count in slanDCs (*A*/*B*), CD1 + DCs (*C*/*D*), and monocytes (*E*/*F*) were evaluated at baseline (BL), 4, 12, and 24 months (M) follow-up of 35 FTY-treated RRMS patients. In parallel absolute cell count of CD4+ T cells, proportion of CD39 + FoxP3+ Treg cells and CD154 + IL17+ Th17 cells was examined (*G*–*I*). Ratio of Treg/Th17 is depicted (*K*). **b** Activation and maturation markers of APC during FTY treatment. Expression of activation and co-stimulatory surface markers were analyzed at baseline (BL), 4, 12, and 24 months (M) in FTY-treated RRMS patients in slanDCs (*A*–*D*), CD1 + DCs (*E*–*H*), and monocytes (*I*–*M*). Mean values ± SEM are presented. Bonferroni’s correction for compared pairs was used for multiple testing. *Asterisks* indicate a statistically significant difference (*p < 0.05, **p < 0.01, ***p < 0.001)

In FTY-treated RRMS patients, there was a relative and absolute increase of slanDCs frequency starting after treatment initiation and during follow-up of 24 months (Fig. 1a (A/B)). In contrast, CD1 + DCs and monocytes increased in relative but not in absolute frequency (Fig. 1a (C–F)). While CD4+ T cell levels significantly decreased from the start of treatment on (Fig. 1a (G)), there was a gradual reduction of the proportion of CD154+ IL17+ Th17 cells over time. The proportion of CD39+ FoxP3+ Treg cells gradually increased (Fig. 1a (H/I)). Therefore, an increase in the ratio of Treg/Th17 could be observed during the first year of FTY treatment (Fig. 1a (K)).

### Decrease of activation/maturation markers and pro-inflammatory cytokine secretion in slanDCs during long-term FTY treatment

During FTY treatment, a decreased ex vivo surface expression of CD83, CD150, and HLADR on APCs over the 24 months could be described (Fig. 1b). All DC subsets showed an increase of CD86 (Fig. 1b (C/G)), which remained unchanged in monocytes (Fig. 1b (L)). CD80 expression was downregulated in slanDCs but not in CD1 + DCs and monocytes (Fig. 1b (D/H/M)). CD40 was unaffected in all investigated APC subsets (data not shown).

SlanDCs of untreated RRMS patients presented with higher levels of expression of IL-1beta, TNF-alpha as well as IL-12 and IL-23 compared to cells from FTY-treated patients (Table 2). In CD1 + DCs from FTY-treated patients, there was no modulation of IL-12 and IL-23 release upon stimulation compared to untreated MS patients (Table 2). Production of IL-6 by slanDCs and CD1 + DCs was lower in FTY-treated patients compared with controls, but differences did not reach statistical significance (Table 2). In monocytes from FTY-treated patients, release of IL-1beta and TNF-alpha was also inhibited, whereas IL-6 secretion was unchanged (Table 2).

**Table 2:** Cytokine release of APC during FTY treatment

APC subtype	Cytokine	MS CTRL	MS FTY	*p* value
slanDC	IL-1beta	13,992.8 (+/−3452.7)	3762.3 (+/−1773.8)	<0.01
	IL-6	68,729.8 (+/−22,461.7)	52,339.4 (+/−23,723.3)	n.s.
	TNF-alpha	41,189.0 (+/−7526.0)	18,302.1 (+/−6363.5)	<0.05
	IL-12	484.9 (+/−99.3)	179.7 (+/−44.6)	<0.05
	IL-23	3298.2 (+/−990.9)	1304.6 (+/−326.2)	<0.05
CD1 + DC	IL-1beta	405.1 (+/−48.3)	255.7 (+/−57.4)	<0.05
	IL-6	7556.1 (+/−3401.0)	5270.4 (+/−2260.8)	n.s.
	TNF-alpha	1190.7 (+/−289.7)	653.1 (+/−19.6)	<0.05
	IL-12	166.1 (+/−54.0)	182.3 (+/−42.9)	n.s.
	IL-23	213.2 (+/−15.9)	188.7 (+/−28.8)	n.s.
Monocytes	IL-1beta	6517.8 (+/−1073.1)	4300.2 (+/−620.1)	<0.05
	IL-6	110,065.0 (+/−17,602.5)	106,537.0 (+/−23,730.0)	n.s.
	TNF-alpha	9018.8 (+/−2753.8)	4841.4 (+/−809.6)	<0.05

Sorted slanDCs, CD1 + DCs, and monocytes of each ten untreated (MS CTRL) and FTY-treated (MS FTY) RRMS patients were stimulated to induce and analyze cytokine release. Mean values of cytokines in picograms per milliliter are presented, and *p* values indicate level of statistical significance

*n.s.* not statistically significant

### Different in vitro modulation of activation markers and cytokine secretion by FTY and FTYP in different APCs

Evaluating effects of FTY or FTYP in vitro and sorted APC of healthy controls were co-incubated with FTY and FTYP: SlanDCs, but not CD1 + DCs, decreased their CD83 expression in response to FTY and FTYP (Table 3). Upregulation of activation marker CD150 in treated monocytes was significantly impaired after FTY or FTYP co-incubation compared with untreated controls (Table 3). No significant alteration in HLADR, CD86, CD80, or CD40 expression could be shown in any investigated cells after FTY or FTYP co-culture in vitro (Table 3).

**Table 3:** Cytokine release and activation/maturation markers after FTY or FTYP in vitro

APC subtype	Cytokine	Without	FTY	*p* value	FTYP	*p* value
slanDC	IL-1beta	26,753.7 (+/−13,156.6)	750.8 (+/−277.3)	<0.05	6835.1 (+/−2634.6)	<0.05
	IL-6	69,634.3 (+/−18,883.2)	7180.5 (+/−1748.1)	<0.05	46,643.9 (+/−8215.2)	<0.05
	TNF-alpha	18,837.0 (+/−9012.4)	1576.9 (+/−267.4)	<0.05	5960.4 (+/−2131.8)	<0.05
	IL-12	637.7 (+/−141.8)	92.2 (+/−19.5)	<0.01	312.8 (+/−153.3)	n.s.
	IL-23	4423.2 (+/−2068.8)	136.9 (+/−29.9)	<0.05	1919.4 (+/−644.6)	<0.05
CD1+ DC	IL-1beta	624.4(+/−282.8)	266.2 (+/−37.1)	<0.05	284.3 (+/−15.7)	<0.05
	IL-6	6889.3 (+/−1367.2)	1259.1 (+/−91.5)	<0.01	5413.8 (+/−1100.4)	n.s.
	TNF-alpha	2203.0 (+/−1140.7)	933.9 (+/−387.3)	<0.05	468.6 (+/−37.9)	<0.05
	IL-12	74.8 (+/−20.5)	66.4 (+/−6.6)	n.s.	66.3 (+/−7.2)	n.s.
	IL-23	250.7 (+/−36.1)	251.2 (+/−61.7)	n.s.	189.1 (+/−22.9)	n.s.
Monocytes	IL-1beta	8670.9 (+/−4065.2)	4902.8 (+/−1932.1)	<0.05	599.8 (+/−113.4)	<0.05
	IL-6	105,741.0 (+/−24,004.5)	31,584.8 (+/−11,302.5)	<0.05	32,223.6 (+/−8824.4)	<0.05
	TNF-alpha	2050.2 (+/−622.3)	1149.2 (+/−345.7)	n.s.	705.1 (+/−164.8)	<0.05
APC subtype	Surface marker	Without	FTY	*p* value	FTYP	*p* value
slanDC	CD83	866.6 (+/−88.9)	426.8 (+/−34.5)	<0.05	477.5 (+/−63.7)	<0.05
	HLADR	243.1 (+/−40,9)	254.7 (+/−56,7)	n.s.	300.0 (+/−73,5)	n.s.
	CD86	681.6 (+/−115.1)	571.6 (+/−105.9)	n.s.	670.3 (+/−105.4)	n.s.
	CD80	896.5 (+/−42.3)	812.5 (+/−39.1)	n.s.	906.5 (+/−63.7)	n.s.
	CD40	510.0 (+/−72.9)	573.4 (+/−100.1)	n.s.	542.5 (+/−90.2)	n.s.
CD1 + DC	CD83	720.9 (+/−135.9)	587.8 (+/−128.7)	n.s.	707.0 (+/−111.3)	n.s.
	HLADR	405.3 (+/−94.0)	418.0 (+/−63.5)	n.s.	327.4 (+/−75.7)	n.s.
	CD86	810.8 (+/−189.2)	703.3 (+/−64.8)	n.s.	893.4 (+/−197.2)	n.s.
	CD80	632.8 (+/−61.7)	588.5 (+/−45.7)	n.s.	601.5 (+/−23.6)	n.s.
	CD40	556.5 (+/−44.3)	650.0 (+/−75.6)	n.s.	578.6 (+/−50.8)	n.s.
Monocytes	CD150	253.2 (+/−23.8)	80.7 (+/−8.3)	<0.05	106.8 (+/−5.9)	<0.05
	HLADR	131.1 (+/−17.7)	177.0 (+/−36.5)	n.s.	109.1 (+/−18.5)	n.s.
	CD86	295.8 (+/−27.0)	352.0 (+/−36.7)	n.s.	243.6 (+/−25.9)	n.s.
	CD80	165.8 (+/−29.6)	171.3(+/−15.6)	n.s.	162.8 (+/−22.4)	n.s.
	CD40	395.0 (+/−49.7)	459.5 (+/−58.2)	n.s.	332.1 (+/−47.7)	n.s.

Sorted slanDCs, CD1+ DCs, and monocytes of eight healthy donors were stimulated in the absence (without) or presence of 30 ng/ml FTY or FTYP. Release of pro-inflammatory cytokines and expression of surface maturation/activation markers was then evaluated. Mean values of cytokines in picograms per millliliter or MFI of surface expression are presented, Bonferroni’s correction was used for multiple testing, and *p* values indicate level of statistical significance

*n.s.* not significant

In vitro addition of FTY and FTYP reduced IL-1beta, IL-6, TNF-alpha, IL-12, and IL-23 secretion in slanDCs compared with untreated controls (Table 3). Interestingly, FTY exerted a stronger suppressive effect than FTYP (Table 3). In CD1+ DCs, only IL-1beta and TNF-alpha but not IL-12 and IL-23 cytokine production was reduced by FTY and FTYP in vitro (Table 3). IL-6 was inhibited significantly only by FTY (Table 3). Both FTY and FTYP significantly inhibited pro-inflammatory in vitro cytokine release of IL-1beta, IL-6, and TNF-alpha in monocytes compared with untreated controls (Table 3).

### SlanDC are modulated by S1P in healthy donors but not FTY-treated patients

**Fig. 2:**
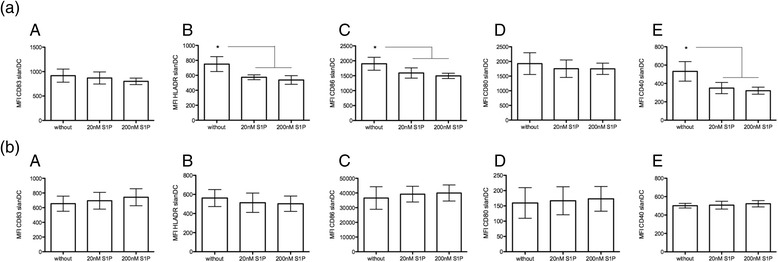
Activation and maturation marker after S1P. **a** Sorted slanDCs (*A*–*E*) of healthy donors were cultured in the absence (without) or presence of 2 or 200 nM S1P. Expression of activation and maturation surface markers was analyzed. **b** Sorted slanDCs (*A*–*E*) of FTY-treated MS patients were cultured in the absence (without) or presence of 20 or 200 nM S1P. Expression of activation and maturation surface markers was analyzed. Mean values ± SEM of eight individual experiments are presented. Bonferroni’s correction was used. *Asterisks* indicate statistically significant difference (*p < 0.05)

In similar in vitro experiments, S1P modulated the expression of HLADR, CD86, and CD40 but not CD83 or CD80 on slanDCs of healthy donors (Fig. 2a) or of surface markers on CD1+ DCs and monocytes of healthy controls (data not shown). Interestingly, in further ex vivo analyses, sorted slanDCs of FTY-treated patients that were cultured in the presence or absence of 20 or 200 nM S1P did not present any additional changes in surface expression of activation or maturation markers (Fig. 2b). Neither sorted CD1+ DC nor sorted monocytes of FTY-treated patients were affected with respect to the expression of surface markers after culture in the presence of S1P (data not shown). There was no impact of 20 or 200 nM S1P on pro-inflammatory cytokine release in sorted slanDCs, CD1+ DCs, or monocytes of healthy controls or FTY-treated patients in vitro (data not shown).

### Modulation of DC-dependent T cell proliferation and polarization in vitro and in FTY-treated patients without any direct effects on T cells

**Fig. 3:**
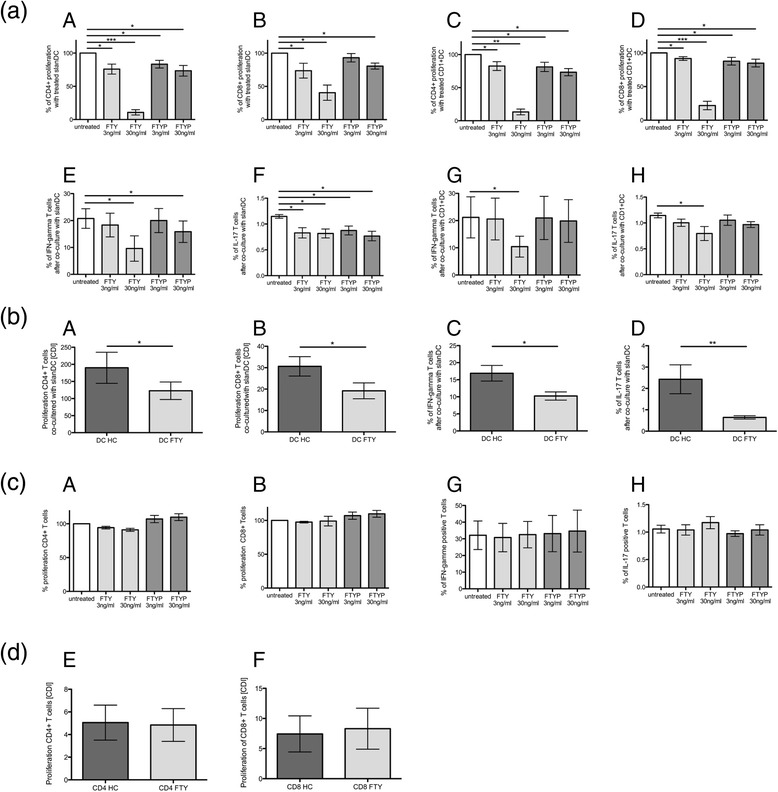
DC-dependent and independent T cell proliferation and programming. **a** SlanDCs or CD1+ DCs of healthy donors were pretreated with different concentrations of FTY or FTYP and co-cultured with allogeneic CFSE-labeled CD4+ or CD8+ T cells. DC-depending proliferation was calculated by CFSE-incorporation by flow cytometry and quantified by CDI. Proliferation after pretreatment is depicted in proportion to proliferation without pretreatment (*A*–*D*). SlanDCs or CD1+ DCs of healthy donors were pretreated with different concentrations of FTY or FTYP and co-cultured with allogeneic naïve CD4+ T cells. T cells were then analyzed regard their differentiation into pro-inflammatory IFN-gamma-expressing Th1 cells (*E*/*F*), IL-17-expressing Th17 cells (*G*/*H*). Mean values ± SEM of eight individual experiments are presented. **b** In addition slanDCs of FTY-treated MS patients (DC FTY) and of healthy controls (DC HC) were co-cultured with allogeneic CFSE labeled CD4+ or CD8+ T cells of the same donor. DC-dependent proliferation was calculated by CFSE-incorporation by flow cytometry and quantified by CDI (*A*/*B*). SlanDCs of FTY-treated RRMS patients (DC FTY) and healthy controls (DC HC) were co-cultured with allogeneic naïve CD4+ T cells of the same donor. T cells were then analyzed regard their differentiation into Th1 cells (*C*) and Th17 cells (*D*). Mean values ± SEM of five different donors are presented. **c** DC-independent T cell proliferation and polarization. FTY- and FTYP-treated CFSE-labeled CD4+ or CD8+ T cells of healthy donors were stimulated with human anti-CD3/CD28. Proliferation was calculated by CFSE-incorporation by flow cytometry and quantified by CDI (*A*–*B*). Polarization of FTY- and FTYP-treated naïve CD4+ T cells of healthy donors into IFN-gamma-expressing Th1 cells or IL-17-expressing Th17 cells are depicted (*G*–*H*). Mean values ± SEM of six individual experiments are presented. **d** In addition, proliferation of CD4+ or C8+ T cells of FTY-treated patients (CD4 FTY, CD8 FTY) compared to healthy controls (CD4 HC, CD8 HC) after anti-CD3/CD28 stimulation was evaluated (*A*/*B*). *Asterisks* indicate a statistically significant difference (*p < 0.05, **p < 0.01, ***p < 0.001)

In vitro pretreatment with FTY and, to a lower extent, FTYP of slanDC and CD1 + DC demonstrated a decrease in DC-dependent T cell proliferation in a dose-depending manner in CD4+ T cells rather than in CD8+ T cells (Fig. 3a (A–D)). FTY and FTYP pretreated sorted slanDCs and CD1 + DCs were significantly inhibited in their ability to promote their typical differentiation of naïve CD45RA+ CD4+ T lymphocytes into pro-inflammatory IFN-gamma-expressing Th1 cells or IL-17-expressing Th17 cells (Fig. 3a (E–H)), whereas pretreatment of CD1 + DCs with FTYP showed no significant influence (Fig. 3b (G/H)). Differentiation toward anti-inflammatory Th2 cells releasing IL-4 was not modulated by FTY or FTYP pretreatment of slanDC or CD1 + DC in vitro (data not shown).

These results were mirrored in slanDCs of FTY-treated patients ex vivo: SlanDCs of FTY-treated patients induced less proliferation in allogenic CD4+ or CD8+ T cells compared to slanDCs from healthy controls (Fig. 3b (A/B)). Compared to healthy controls, slanDCs of FTY-treated patients were impaired in their induction of differentiation of naïve CD45RA+ CD4 T cells into pro-inflammatory IFN-gamma-releasing Th1 cells or IL17-releasing Th17 cells (Fig. 3b (C/D)), whereas differentiation into Th2 cells was again unaffected (data not shown).

In contrast, neither FTY nor FTYP directly affected CD4+ or CD8+ T cell proliferation in vitro (Fig. 3c (A/B)). FTY or FTYP did not directly affect any polarization of naïve CD45RA+ CD4+ T lymphocytes into IFN-gamma-expressing Th1 cells, IL-17 releasing Th17 (Fig. 3c (C/D)), or IL-4 releasing Th2 cells (data not shown). Furthermore, CD4+ and CD8+ T cells of FTY-treated patients demonstrated similar proliferative capacity after CD3/CD28 stimulation compared to healthy controls (Fig. 3d (A/B)).

### FTY exerts differential effects on phagocytic function, but not on apoptosis of APC

**Fig. 4:**
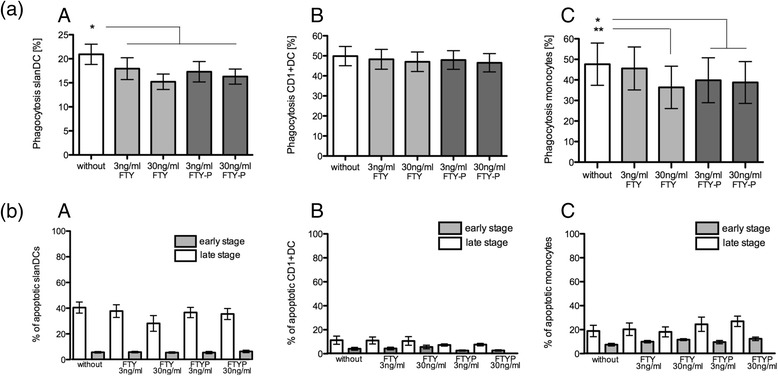
Phagocytosis and apoptosis after FTY. **a** SlanDCs (*A*), CD1 + DCs (*B*), and monocytes (*C*) were treated with different concentrations of FTY or FTYP before phagocytosis of carboxylate-modified yellow–green fluorescent beads was evaluated. Mean values ± SEM of eight individual experiments are presented. Bonferroni’s correction was used. *Asterisks* indicate a statistically significant difference (*p < 0.05, **p < 0.01). **b** SlanDCs (*A*), CD1+ DCs (*B*), and monocytes (*C*) were treated with different concentrations of FTY or FTYP for 24 h (*A*–*C*) or 48 h (data not shown). Afterwards, apoptosis at early (*gray bar*) and late stage (*white bar*) was evaluated. Mean values ± SEM of eight individual experiments are presented

In slanDCs and monocytes, but not in CD1+ DCs, phagocytic capacity was significant and dose depending inhibited by FTY and FTYP (Fig. 4a (A–C)). In contrast to phagocytic function, neither FTY nor FTYP increased apoptosis in any investigated APCs within all investigated time intervals (Fig. 4b (A–C)).

## Discussion

A growing number of studies highlight the relevance of sphingolipids and their related pathways that regulate innate immunity [13, 17, 18]. Due to their characteristic properties of antigen uptake and antigen presentation to T cells, DCs are particular key players in balancing tolerogenic and immunogenic immune responses [5]. Many studies hint at the importance of APCs, and particularly DCs, in the pathogenesis of MS by virtue of the initiation and perpetuation of T cell responses in periphery as well as in the CNS [3, 6–9]. Growing evidence supports the concept of distinct modulation of innate immune cells in S1PR-focused therapies beyond their effects on lymphocyte recirculation [14, 17, 18].

During long-term follow-up, the relative number of peripheral slanDCs, CD1+ DCs, and monocytes increased in FTY-treated patients. These effects are in line with the redistribution of peripheral lymphocytes during FTY treatment. But the absolute number of slanDCs also increased. Expression of S1PR1-S1PR4 on monocyte-derived DC surfaces has been investigated, and S1PR4 appears to be one of the dominant receptors subtypes in that environment [19]. The impact of FTY and S1PR agonists on APC migration are contradictory and seem to depend on maturation and differentiation [18, 22, 23]. Some reports have shown an increase in peripheral DC numbers in mice, combined with a decrease in the number of DCs in lymph nodes, thereby suggesting a downregulation of CCR7—but not S1PR1—during FTY treatment [18, 20, 22–24]. Additionally, reduced migration of DCs in the presence of certain chemokines after FTY has been shown, and this indicates the possibility of inhibited DC migration into the CNS in inflammatory conditions such as those seen in active MS [20, 24]. Different studies demonstrated significant expression of S1PR1-4 on human and murine DC subtypes [16, 23, 25]. Systemic FTY administration leads to a decrease in expression of surface adhesion molecules and chemokine receptors including CCR7, which are essential for a variety of migratory processes [20, 23]. S1PR agonism impaired DCs in activation and differentiation, which is important to upregulate adhesion molecules and chemokine receptors as well [16, 23]. Reduced expression of these markers contributes to a decreased homing of DCs into lymphoid organs, but into inflamed tissues. These findings indicate that DC migration is additionally controlled by S1PRs and affected by S1PR-targeted therapies that account for increase in peripheral absolute DC count in our and recent study [20, 23]. Further studies are needed to investigate the exact effect of FTY and its homologues on migration of slanDCs or other dendritic cells.

Due to the established effects of FTY on lymphocyte recirculation, CD4+ T cell counts decreased in our FTY-treated patients. But among CD4+ T cells exposed to FTY, the proportion of Th17 cells was reduced, while Treg cell numbers increased, thereby increasing the Treg/Th17 cell ratio of cells. These data are in line with previous reports of FTY use in patients that demonstrated a re-balanced distribution of T cells by virtue of decreased levels of effector T cells and increased levels of Treg cells [26–28]. However, a direct or DC-independent impact of FTY on T cell polarization or proliferation in vitro could not be demonstrated [29]. As APCs, and particularly DCs, are the most potent inducers and regulators of T cell responses, the potential modulation of DC function by FTY could be very powerful.

Upon activation and antigen uptake, DCs maturate, differentiate, and upregulate expression of surface markers such as CD83 or CD150 and co-stimulatory molecules including HLA-DR, CD86, CD80, or CD40. During FTY treatment in our patients, chiefly slanDCs, but also CD1 + DCs and monocytes, failed to maturate and express the co-stimulatory marker HLA-DR. This is in line with previous data [16, 21].

APCs that fail to differentiate or increase expression of their co-stimulatory markers have impaired antigen presentation and T cell activation properties, which may lead to decreased induction of pro-inflammatory Th1/Th17 cell responses. CD86 is upregulated during the differentiation process on APCs, and there are some reports suggesting its relevance for induction of tolerance mechanisms in a range of immunological diseases [30]. In our analysis, the expression of CD86 was increased in slanDCs and CD1+ DCs. These data are in concordance with those previously presented for other DC subsets [14].

Our data suggest distinct but straightforward modulation of APC function by FTY treatment. In the literature, data on DC surface markers during FTY treatment have provided mixed results. Some studies did not find any impact on surface expression, particularly in in vitro studies [15, 16]. Other findings are in line with our results [14, 24, 31]. Certainly, in our analyses, impact of FTY and FTYP on expression of co-stimulatory and maturation markers was more pronounced in ex vivo analyses compared to in vitro investigations. These differences may be explained by the relatively shorter exposure time for in vitro FTY and FTYP compared with in vivo experiments. Human DCs present a pattern of selective expression of S1PR1-4 with highest levels of S1PR4 on immature DCs [16, 25]. Upon maturation, especially S1PR1 is upregulated whereas S1PR4 is slightly decreased and S1PR2-3 are almost unaffected [16, 32]. After FTY exposure, expression of S1PR1 and S1PR4 is reduced already after a short time period in human DCs [16]. Further reports demonstrated direct modulation of inflammatory and T cell stimulatory characteristics via S1PR4 [33, 34]. FTY acts as unselective S1PR agonist affecting S1PR1-5. Interestingly, compared to FTY, findings on S1PR expression differ after treatment with selective S1PR agonists in human DCs [20]. These results indicate that impact of FTY on DCs is mediated by regulation of specific receptor expression profiles as well as direct intracellular signaling after S1PR agonism.

Interestingly, addition of S1P decreased surface expression of co-stimulatory molecules in slanDCs of healthy donors, leaving other APS subtypes unaffected. However, in FTY-treated patients, S1P-dependent modulation of surface markers on slanDCs was abrogated. These data further highlight slanDCs as distinct targets in modulation of S1P pathways via S1PR, and distinct from other APCs.

In addition to modulation of surface markers, further FTY-stimulated inhibition of pro-inflammatory cytokines was detected in slanDCs, CD1+ DCs, and monocytes both in vitro and ex vivo. Previous reports have shown a similar pattern of decrease of pro-inflammatory cytokines by fingolimod in murine bone marrow-derived DCs and induction of anti-inflammatory macrophages [14, 15, 19, 24]. Furthermore and comparable to other reports in mice, phagocytic capacity was inhibited in FTY-treated slanDCs and monocytes in our study [15]. These results suggest that FTY impairs important DCs capabilities, which are important for antigen uptake and processing as well as the pro-inflammatory destructive demyelinating process of the CNS in MS pathology [15, 35–38].

IL-12 and IL-23 are known as essential regulators in DC-depending Th1 and Th17 differentiation and programming. The relevance of sphingolipids in IL-12 and IL-23 secretion in DCs and decreased expression of IL-12 in bone marrow-derived DCs and monocytes-derived DCs after FTY has been already reported [15, 16, 18, 39–41]. However, slanDCs are one of the most potent inducers of Th17 and Th1 cells [42, 43]. Our data demonstrate significant inhibition of IL-12 and IL-23 secretion after FTY exposure in vitro and ex vivo in slanDCs, but not in other DCs. In addition, we were able to demonstrate that FTY- or FTYP-treated slanDCs had reduced capacity to induce Th17 and Th1 cells as well as T cell proliferation in vitro. For the first time, these results could be confirmed in ex vivo experiments using slanDCs of FTY-treated patients. Th17 cells are known to be reduced in peripheral blood and CNS during FTY treatment [26–28]. Previous studies presented that S1PR4 on DCs is critically involved in promotion of Th17 cells [33].

We suggest that modulation of slanDCs by FTY treatment promotes the reduction of pro-inflammatory T cell activation and proliferation in MS. Effects on CD1+ DCs were less pronounced. Other studies have demonstrated a decrease in polarization of Th1 cells, but promotion of Th2 cells by FTY-treated monocytes-derived DCs [16, 18, 19, 44]. Nevertheless, we could not identify increased activation of IL-4-producing Th2 cells by FTY-treated slanDCs or CD1+ DCs in vitro or ex vivo.

## Conclusions

Our data demonstrate that S1PR-targeted therapies can act additionally as immunomodulators that affect the innate immune system beyond their established prominent effects on lymphocyte trafficking. Our results highlight that FTY can modulate essential DC-dependent pro-inflammatory T cell-mediated pathways in MS immunology, which could additionally contribute to the efficacy of FTY. These effects appear to develop over the first year of treatment, which is on a slower timescale than the instant effect of FTY on lymphocyte distribution. Other mechanisms of action are probably as well of importance because there is no direct link between absolute lymphocyte counts and therapeutic efficacy [45, 46]. In summary, we present supportive data suggesting innate immune cells as additional important targets of S1PR-directed therapies for MS.

## Abbreviations

APC: Antigen-presenting cells
CDI: Cell division index
CFSE: Carboxyfluorescein-di-acetate-*N*-succinimidylester
CNS: Central nervous system
DC: Dendritic cells
FACS: Fluorescence-activated cell sorting
FCS: Fetal calf serum
FTY: Fingolimod
FTYP: Fingolimod-phosphate
IFN: Interferon
IL: Interleukin
MS: Multiple sclerosis
PBMC: Peripheral mononuclear cells
PBS: Phosphate-buffered saline
PMA: Phorbol myristate acetate
RRMS: Relapsing remitting multiple sclerosis
S1P: Sphingosine-1-phosphate
S1PR: Sphingosine-1-phosphate receptor
Th cells: T helper cells
TNF-alpha: Tumor necrosis factor alpha
Treg cells: T regulatory cells
